# Deciphering the anti-atherosclerotic mechanisms of Danggui-Chuanxiong herb pair: a synergistic approach combining computational prediction and *in vivo* verification

**DOI:** 10.3389/fcvm.2026.1717999

**Published:** 2026-02-10

**Authors:** Yuemeng Sun, Yixing Lu, Fei Ge, Hao Wu, Zhanhong Qian, Jianbei Chen, Hua Hao

**Affiliations:** 1 College of Traditional Chinese Medicine, Inner Mongolia Medical University, Hohhot, China; 2 School of Chinese Medicine, Beijing University of Traditional Chinese Medicine, Beijing, China; 3 School of Chinese Medicine, Hubei University of Chinese Medicine, Wuhan, China; 4 Hubei Shizhen Laboratory, Hubei University of Chinese Medicine, Wuhan, China

**Keywords:** atherosclerosis, Danggui-Chuanxiong, molecular docking, network pharmacology, vascular endothelium

## Abstract

**Introduction:**

Danggui-Chuanxiong is a classic Chinese medicine formulation clinically employed for atherosclerosis (AS) management by modulating lipid metabolism and improving hemodynamic properties.

**Materials and methods:**

Integrated network pharmacology, molecular docking and molecular dynamics simulation approaches were employed to elucidate the material basis and therapeutic mechanisms of DG-CX in AS treatment, and validated by subsequent experiments. The regulatory effects of DG-CX on the c-Abl/YAP signaling pathway were comprehensively assessed through integrated histopathological and molecular analyses.

**Results:**

Network pharmacology analysis identified sterols, stigmasterol, and wallichilide as the principal bioactive constituents of DG-CX. Furthermore, molecular docking validated that VEGFR2 and PTGS2 are the core targets, which are potentially mediated through c-Abl/YAP signaling pathway, consistent with strong binding affinities. Molecular dynamics simulations of the protein-ligand complexes revealed that the PTGS2-FER and VEGFR2-β-sitosterol complexes exhibited stable binding, with favorable hydrogen bonding interactions. Our research results show that DG-CX can reduce the body weight of AS mice, improve lipid metabolism disorders, and upregulate the expression of NO/NOS, AngII/AT1R, and PGI2 (*p* < 0.01), inhibit the expression of VEGFR2 and PTGS2 mRNA (*p* < 0.05 or *p* < 0.01), and the expression of the Interα5β1/c-Abl/YAP pathway.

**Conclusions:**

Validation using network pharmacology, molecular docking, molecular dynamics simulation and *in vivo* studies suggested the efficacy of DG-CX in improving vascular endothelial function and exerting anti-AS effects by inhibiting the c-Abl/YAP signaling pathway.

## 1 Introduction

Atherosclerosis (AS) is a fundamental contributor to various cardiovascular and cerebrovascular pathologies, such as coronary heart disease, hypertension, and dyslipidemia, and is a primary etiological factor for stroke and ischemic heart disease (1). The hallmark pathological feature is the development of yellowish atheromatous plaques, predominantly affecting medium- and large-sized arteries (2, 3), accompanied by inflammatory reactions, endothelial dysfunction, and abnormalities in platelet aggregation (4). Current statistics indicate that AS-related diseases are responsible for up to 20 million annual deaths worldwide, seriously threatening the lives and health of humans (3).Studies have shown that the pathogenesis of AS is closely related to dyslipidemia, inflammatory responses, vascular endothelial damage, and turbulent flow (5, 6). Therefore, reducing inflammation, protecting the function of the vessel endothelium, and regulating the turbulent flow might be one of the effective approaches to combat AS.

**Figure 1:**
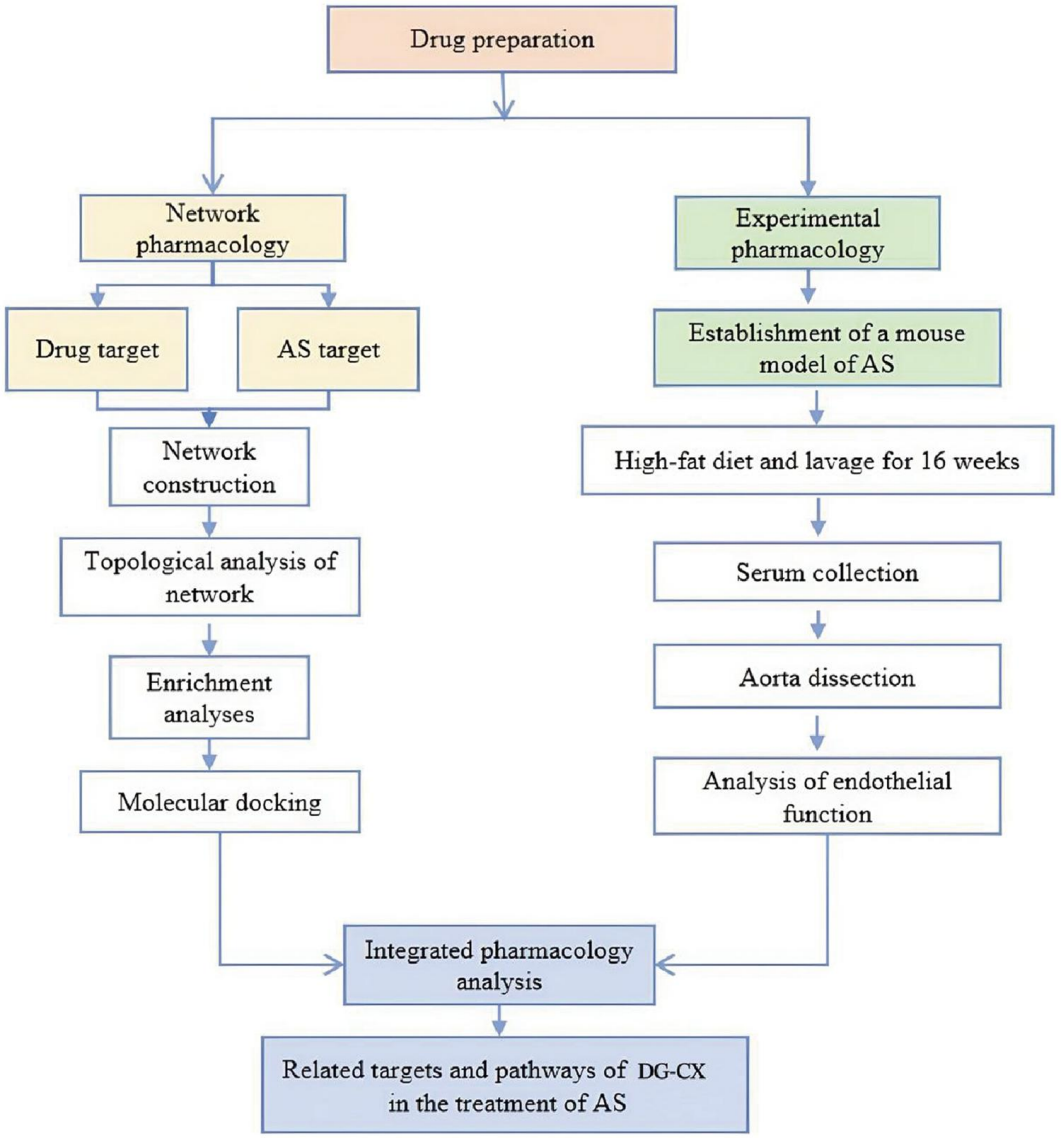
Flowchart of experimental design.

Network pharmacology is a useful tool that reveals the function and behavior of complex biological systems (7). It has been widely used to elucidate the intrinsic relationships between the active ingredients of herbal medicines and therapeutic targets of diseases, and to explore the mechanisms of action of several molecules (8–10). By visualizing and analyzing intermolecular interaction networks and related data, researchers can comprehensively and systematically identify the targets of herbal medicines and assess their effects in diseases (11, 12). An integrated pharmacology strategy of the combination of network pharmacology and polypharmacology was used in this study. Network pharmacology was used in the first phase to elucidate the active constituents of DG-CX and their prospective therapeutic targets and pathways against AS. Next, experiments were designed to validate the mechanism of action of DG-CX in alleviating AS (Figure 1).

DG-CX first appeared in the “Taiping Huimin Hejian Bufang” of the Song Dynasty, in which “Xiong Gui Decoction”, a combination of two Chinese herbs, is commonly used in Chinese medicine as a pair (13). These two herbs have the effects of activating blood circulation, dispersing blood stasis, nourishing the blood, and tonifying the blood (14) and are a classic pair commonly used in Chinese medicine clinics. Increasing evidence from recent investigations demonstrates the multifaceted pharmacological properties of DG-CX, including but not limited to its anti-inflammatory, antioxidant, and anticoagulant activities (15, 16). This combination has been widely used to treat migraine, ischemic stroke, and AS (17–19). DG-CX is known to significantly reduce serum triglyceride (TG), total cholesterol (TC), and low-density lipoprotein-cholesterol (LDL-C) levels, increase high-density lipoprotein-cholesterol (HDL-C) levels, and reduce the coronary atherosclerotic plaque area index in hyperlipidemic rats (20). Our previous study (21) showed that DG-CX could effectively alleviate high-fat diet–induced lipid metabolism disorders in mice, reduce the platelet aggregation rate, and decrease the expression of cell adhesion factor, effectively inhibiting AS. Furthermore (22, 23), DG-CX could improve hemodynamics in rats, lower blood viscosity, and enhance erythrocyte deformability. However, most studies have demonstrated the anti-AS effect in a compound form. Studies focusing solely on the multi-target prevention and treatment of AS using only astragalus and chuanxiong are relatively rare. This combination contains numerous active components, challenging the elucidation of its mechanism of action. Furthermore, the multicomponent and multitarget characteristics of traditional Chinese medicine pose significant impediments to comprehensively evaluating the therapeutic efficacy of DG-CX. Therefore, identifying the active constituents and molecular targets of DG-CX could serve as a viable strategy in developing AS therapeutics.

An integrated approach integrating network pharmacology, molecular docking, molecular dynamics simulations, and experimental validation was used to elucidate the anti-atherosclerotic mechanisms of DG-CX and its clinical potential.

## 2 Materials and methods

### 2.1 Screening potential compounds and targets of DG-CX

The disease-protective effects of DG-CX were investigated using a network pharmacology approach. First, the chemical constituents of DG-CX were retrieved from The traditional Chinese medicine systems pharmacology (TCMSP) database and analytical platform (TCMSP) (24). Active compounds were screened based on oral bioavailability (OB) ≥30% and drug-likeness (DL) ≥0.18 to predict targets. The canonical SMILES structures of these active compounds were obtained from the PubChem database (https://pubchem.ncbi.nlm.nih.gov/) and subsequently submitted to the Swiss Target Prediction (http://www.swisstargetprediction.ch/) platform for target identification (25). Targets with a prediction probability greater than 0 were retained, from which core potential therapeutic targets were ultimately determined.

### 2.2 Atherosclerosis-associated genes screening

Genes exhibiting a relevance score of at least 3 (26)—indicating the strength of association between the gene and AS, with higher scores reflecting greater relevance—were selected for inclusion in the screening from the GeneCards database (27). The relevance score is computed by GeneCards using a term frequency/inverse document frequency algorithm; further details regarding the scoring methodology are available at https://www.genecards.org/Guide/Search#relevance. In summary, an elevated relevance score corresponds to a stronger semantic association between the gene and the search term. A comprehensive list of AS-related genes was subsequently compiled by integrating data from the OMIM and DrugBank databases and removing duplicate entries.

### 2.3 Compound-target network construction and the analysis of potential DG-CX targets against atherosclerosis

The Microbiology Letter (http://www.bioinformatics.com.cn) online platform was used to obtain the intersection target of the active targets and disease targets of the drugs, draw the Venn diagram, and obtain the core targets of drug treatment for AS. After target identification, protein interaction data were acquired from the STRING database and subsequently imported into Cytoscape to construct and visualize the protein–protein interaction (PPI) network.

### 2.4 GO function and KEGG pathway enrichment analyses

Potential therapeutic targets for AS intervention were subjected to functional enrichment analysis on the Metascape platform (http://metascape.org) using Gene Ontology (GO) annotation and Kyoto Encyclopedia of Genes and Genomes (KEGG) pathway analysis. GO term annotation and KEGG pathway enrichment analyses were conducted, followed by visualization of the significantly enriched terms and pathways.

### 2.5 Molecular docking validation of active DG-CX compounds with anti-atherosclerotic targets

The potential core targets identified using network pharmacology were subjected to molecular docking with their associated bioactive ligands using AutoDock Tools 1.5.6 to determine binding affinities and interaction patterns. The crystal structures of the target proteins were downloaded from the RCSB PDB database (https://www.rcsb.org), and ligand structures were downloaded from PubChem (https://pubchem.ncbi.nlm.nih.gov/). The downloaded data was preprocessed using AutoDock Tools 1.5.6. The proteins were hydrogenated and dehydrated, the small molecule ligands were hydrogenated, and the torsionical force was determined, etc. To ensure full binding between the ligands and proteins, the system was prepared to allow the proteins to closely wrap around the ligands. Docking results were analyzed, and representative 3D interaction models of key targets were rendered using PyMOL for structural interpretation. The binding energy of the interaction is lower than −5.0 kcal/mol, demonstrating a strong binding capacity (28–32).

### 2.6 Molecular dynamics simulation

This study employed GROMACS 2022 for molecular dynamics simulations. The protein and ligand were modeled using the AMBER14SB force field and the GAFF2 force field with RESP charges, respectively. The system was solvated with TIP3P solvent and neutralized with 0.15 M NaCl ions (33). The long-range electrostatics were treated using the PME method, and bond lengths were constrained using the LINCS algorithm. After three stages of energy minimization, a 100 ns NPT simulation was conducted at 310K and 1 bar. Finally, RMSD, RMSF, hydrogen bonds, Rg, and SASA were analyzed to evaluate the stability and structural changes of the system.

### 2.7 Reagents

NO detection kit (Nanjing Jianjian Bioengineering Research Institute Co., Ltd., A013-2-1), nitric oxide synthase (NOS) detection kit (Nanjing Jianjian Bioengineering Research Institute Co., Ltd., A014-2), angiotensin II (AngII) antibody (Boisson, stock number bs-2771R, Ltd., AC03215689), angiotensin II type-1 receptor (AT1R) antibody (Boisson, stock number bs-1548R, Ltd., BA05216716), interα5β1 integrin antibody (Boisson, stock No. bs-1832R, lot No. BJ05188610), c-Abl antibody (Boisson, sc56887), p-YAP antibody (Boisson, stock No. bs-1832R, lot No. BJ05188610), YAP antibody (abcam, Beyoncé, stock ab205270), chemiluminescence substrate (Thermo Fisher, item No. QD216029A), methanol (Beijing Chemical Factory, item No. 20160318), TRIzol reagent (Invitrogen, item No. 15596025), RNA reverse-transcription kit (Thermo Fisher, item No. K1622), fluorescence quantitative polymerase chain reaction (PCR) reagent (Invitrogen, item No. A25742, 01076916), DEPC water (Kangwei, item No. 31402), DEPC water (Kangwei, item No. 31402), primers (Invitrogen, Shanghai Synthesis Department), 4% paraformaldehyde (OUBEI, OB10341-1), dry powder of phosphate-buffered saline (PBS; Bryolar BN20220-L), powdered antigen repair solution (Myxin, MVS-0066), DAB color development kit (Myxin, 2109290031H), ready-to-use immunohistochemical staining kit (Myxin, 2108029706C), anhydrous ethanol (Sinopharm, 10009218), xylene (Sinopharm, 10023418), and sodium chloride solution (OUBEI, OB20926) were the chemicals and reagents used in this study.

### 2.8 Drug preparation

**Figure 2:**
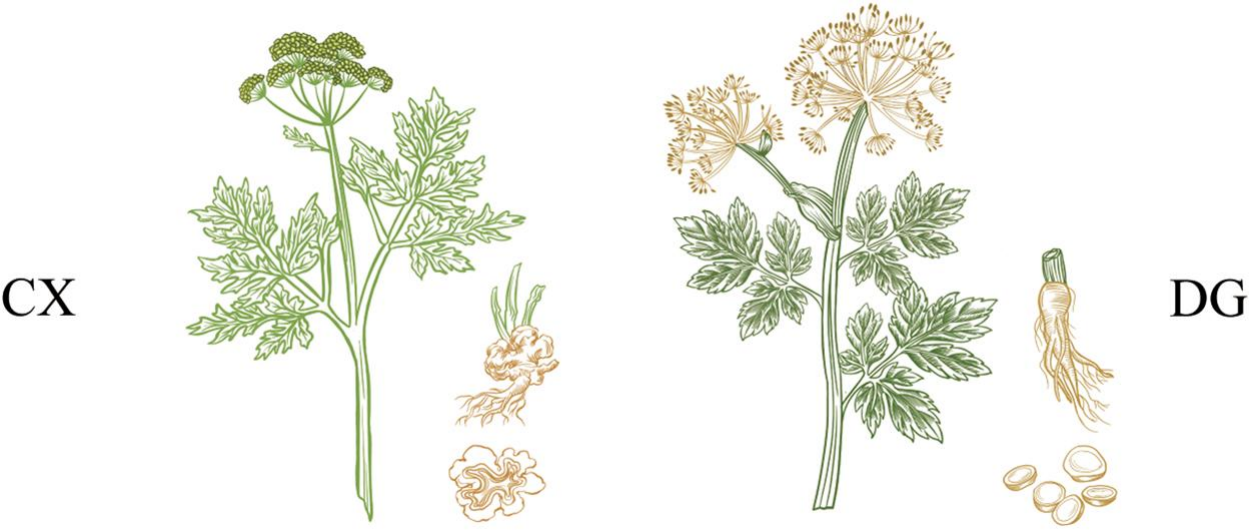
Herbal composition of DG-CX.

DG-CX (21) was purchased from Beijing Tongrentang Jingxi Pharmacy Co., Ltd. (Beijing, China) (Figure 2) (dosage presented in Table 1). The herbal medicines were extracted by soaking the herbs in distilled water for 30 min, followed by boiling over a gentle flame, and then decocting over a mild flame for 30 min. The decoction step was repeated by adding distilled water, removing the drugs, mixing the twice-extracted liquid, and concentrating by evaporation in a water bath to a final concentration of 4.29 g/kg. The extract was then passed through a 200-mesh filter, and all filtrates were refrigerated at 4°C until use. The positive control drug for the experiment was selected as atorvastatin calcium tablets (H20051408, New York, United States) in reference (34). The dosage was converted in accordance with the “Pharmacological Experimental Methodology”, and the dose of statins given per kilogram of mouse weight was expressed in the form of “g/kg”, with a daily dosage of 5 mg/kg. Atorvastatin calcium tablets were vortexted with deionized water to prepare a suspension, which was then stored in a 4℃ refrigerator for future use.

**Table 1:** Dosage of *Angelica sinensis* and *Rhizoma chuanxiong*.

Chinese name	Name of the herb	Medicinal part	Weight (g)	Geographical source of the product
Danggui	*Angelica sinensis* Radix	Root	9	Gansu
Chuangxiong	*Ligusticum striatum* rhizome	Rhizome	24	Sichuan

### 2.9 Animals

Ethical approval for this study was obtained from the Medical Ethics Committee of Inner Mongolia Medical University (Approval No. YKD202403070). Six specific-pathogen-free (SPF)-grade male wild-type C57BL/6J mice and 18 C57BL/6J ApoE knockout (ApoE^−/−^) mice (6–8 weeks old, 20 ± 2 g) were obtained from Beijing Viton Lever Laboratory Animal Technology Co., Ltd [Animal Certification: SCXK (Beijing) 2016-0006]. All animals were housed under SPF conditions, housed in the SPF-grade Laboratory Animal Center of Inner Mongolia Medical University under controlled environmental conditions, which included a temperature of 25 ± 2°C and relative humidity of 40%–60%. All mice were subjected to a 12-h/12-h light/dark cycle and provided access to standard rodent chow and water *ad libitum* throughout the experimental period.

### 2.10 Experimental modeling and intervention

A high-fat diet–induced mouse model of AS was established for this study. Following a one-week adaptive feeding period, ApoE^−/−^ mice were randomly assigned to three groups (*n* = 6) based on their body weight using a random number table: the Atherosclerosis Model group (AS), the Danggui-Chuanxiong group (DG-CX), and the Atorvastatin Calcium group (X). This stratified randomization ensured no significant difference in initial body weight among the groups. All were fed a high-fat diet (15% lard + 2% cholesterol + 0.05% bile acids) to facilitate AS plaque formation. At nine o'clock every morning, the DG-CX group was administered a 4.29 g/kg/day dose of DG-CX intragastrically, and the X group was given a 5 mg/kg/day dose of atorvastatin calcium tablets intragastrically for 16 weeks. Normal C57BL/6J mice served as the control (NC group, *n* = 6) and were fed standard chow and an equal volume of distilled water for 16 weeks.

### 2.11 Serum biochemistry analysis

At the end of the 16th week (35), all mice were fasted overnight and anesthetized with sodium pentobarbital. The blood from the eyeballs was collected, and the serum was used for further evaluation. The serum levels of TC, TG, LDL-C, and HDL-C were measured by a vertical automatic blood biochemical analyzer (7080, HITACHI, Shanghai, China). Aortic vessels were stripped, and the images were photographed using a microscope. The vessels were first fixed in 4% paraformaldehyde. Next, the frozen tissues were cut into 10-µm-thick sections, stained with hematoxylin and eosin, and photographed using a light microscope. The remaining vascular tissues were stored at −80°C until subsequent use.

### 2.12 Oil red O staining analysis

Frozen aortic vessels were cut into 10 µm slices (CM 1950, Leica, Germany), air-dried, then stained with Oil Red O (G1261, Solarbio, China) according to the manufacturer's instructions. To evaluate the effect of drug administration, the ORO-stained slides were observed under a positive fluorescence microscope (DM4B, Leica, Germany) (100×), marked, and then assessed with Image-Pro Plus version 6.0 software (Media Cybernetics, Maryland, United States). The histological sections were assessed independently by two investigators who were blinded to the group assignments, in order to minimize observational bias.

### 2.13 Enzyme-linked immunosorbent analysis

Mouse vascular tissue supernatants were placed at room temperature (approximately 25°C) for 20 min, and NO and NOS levels in vascular tissues were determined following the instructions in the corresponding ELISA kits. NO and NOS levels in vascular tissues were calculated using the following equation:

$$NO_{Content}(\mu mol/g\;prot)=\frac{A_{Determination}-A_{Blank}}{A_{Standard}-A_{Blank}}\times C_{Standard}\times N\;÷\;Cpr$$

Note: C represents the standard concentration (20*μ*mol/L); N is the dilution factor used in the sample pre-treatment process before measurement, with tissue being 2; Cpr is the protein concentration of the tissue homogenate [gprot/L (prot refers to protein)]; V is the volume of the extraction solution added when extracting the sample (L); W is the mass of the sample weighed (g).

$$NOS_{Vitality}(\mu /g\;prot)=\frac{A_{Determination}-A_{Blank}}{\varepsilon }\times \frac{V_{Total}}{V_{Sample}}\times \frac{1}{d\times T}\times N÷Cpr$$

Note: Vtotal: Total volume of the reaction solution, (a + 2.41) ml; Vsample: Sample volume, (a) ml; d: Optical path for color measurement, cm; T: Reaction time, 15 min; ε: Absorbance extinction coefficient of the color-forming substance, 38.3 × 10^; Cpr: Protein concentration of the tissue homogenate, mgprot (prot refers to protein).

### 2.14 Real-time fluorescence quantitative PCR analysis

Total RNA from aortic vascular tissues was extracted using TRIzol reagent, and cDNA synthesis was performed following the instructions in Invitrogen's Reverse-Transcription kit. Real-time fluorescence quantitative PCR experiments were performed using an ABI StepOnePlus fluorescence quantitative PCR instrument. The PCR reaction system is 20 μl: including 10 μl of PowerUPTM SYBRTM Green premix, 0.4 μl each of the upstream and downstream primers (concentration 10 μm), 2 μl of cDNA (cDNA stock diluted 10 times), and 7.2 μl of ddH2O. After diluting the cDNA obtained from the previous experiment by 10 times, a real-time fluorescence quantitative PCR experiment was conducted. The reaction conditions are as follows: amplification conditions are at 95℃ for 10 min, denaturation at 95℃ for 5 s, and at 60℃ for 30 s, totaling 40 cycles. At the end of the reaction, the lysis and amplification curves were analyzed after amplification based on the mouse internal reference gene and converted to CT values, ΔCT = CT (target) – CT (β-actin), ΔΔCT = ΔCT (model group) – ΔCT (normal group), and the relative content of RQ mRNA in the target = 2^–ΔΔC^T. The primer and internal reference sequences are shown in Table 2.

**Table 2:** Primer sequence and internal reference sequence.

Gene name	Forward primer (5′-3′)	Reverse primer (5′-3′)
PTGS2	CCTCGTCCAGATGCTATCTTTG	GGCTTCCAGTATTGAGGAGAAC
VEGFR2	GTCCGAATCCCTGTGAAGTATC	GTGAGTTCATCGCCAACAATC
PGI2	CCTCCATCCATCTCTCTGTCTAA	AGAGGAGAGCAGACACTCTAAC
Interα5β1	GGCTATGTCACCGTCCTTAAT	CTAGCCCATCTCCATTGGTATC
c-Abl	GGAGTATTGCTCTGGGAGATTG	GCTCCATGCGGTAGTCTTT
YAP	GAAAGGGCTCTAGTGGGTAAAG	AAATCAGGCTAAGGGAAGTAAGG
GAPDH	AACAGCAACTCCCACTCTTC	CCTGTTGCTGTAGCCGTATT

### 2.15 Immunohistochemistry analysis

To determine AngII and AT1R levels, the frozen sections were treated with sodium citrate at 95℃ for 10 min and cooled to room temperature. Goat serum working solution was added and incubated for 10 min at room temperature. Next, the primary antibody (AngII and AT1R antibodies diluted with PBS in a 1:280 and 1:50 ratio, respectively) was added and incubated overnight in a wet box at 4℃. Next, the secondary antibody was added and incubated for 1 h at room temperature in the dark. The sections were dehydrated using an increasing ethanol gradient and then treated with xylene I and xylene II for 10 min for transparentization. Lastly, the slices were fixed with neutral gum, sealed, and stored.

### 2.16 Immunofluorescence analysis

To determine the expression of interα5β1 (ITGA5/ITGB1), c-Abl (ABL1), and YAP (YAP1), the frozen tissue sections were processed for antigen retrieval by incubation in preheated sodium citrate buffer (10 mM, pH 6.0) at 95°C for 10 min. After heat treatment, the slides were cooled to room temperature for over 30 min to ensure proper epitope exposure for subsequent antibody binding. Goat serum working solution was added to the sections and incubated for 10 min at room temperature. Next, the primary antibodies (interα5β1, c-Ab l, and YAP antibodies were diluted with PBS in a 1:100, 1:50, and 1:50 ratio, respectively, were added to the sections and incubated overnight in a wet box at 4°C. The fluorescent secondary antibody was diluted with PBS in a 1:200 ratio, added dropwise to the surface of the frozen sections to cover the tissues, and incubated at room temperature and away from light for 1 h. Lastly, the sections were sealed with an anti-fluorescence quenching sealer containing DAPI.

### 2.17 Statistical analysis

SPSS 22.0 statistical software was used for data analysis. Data are expressed as mean ± standard deviation (`x ± s), after ensuring that all data were normally distributed and satisfied the homogenity of variance. One-way ANOVA was used to compare multiple groups. The LSD method was used to compare the differences between the groups when the variance was homogeneous. The Games-Howell method was used to compare differences between groups when the variance was not uniform. A value of *p* < 0.05 indicated that the difference was statistically significant.

## 3 Results

### 3.1 Potential compounds and targets of DG-CX

A total of 10 DG-CX compounds were retrieved from the TCMSP database and combined with references (36) (Table 3), and their main active components included sterols, stigmasterols, cyclodextrins and ferulic acid, etc. After screening, merging and deduplication of the targets obtained by TCMSP and Swiss Target Prediction, 232 related drug targets were finally obtained.

**Table 3:** Information on the active ingredients of DG-CX.

Information Source	Number (Mol ID)	Active ingredient (molecule name)	OB (%)	DL
*Angelica sinensis* Radix	MOL000358	β-sitosterol	36.91	0.75
*Angelica sinensis* Radix	MOL000449	Stigmasterol	43.83	0.76
*Angelica sinensis* Radix	MOL000360	FER	39.56	0.06
*Ligusticum striatum* Rhizoma	MOL000359	Sitosterol	36.91	0.75
*Ligusticum striatum* Rhizoma	MOL002135	Myricanone	40.6	0.51
*Ligusticum striatum* Rhizoma	MOL001494	Mandenol	42	0.19
*Ligusticum striatum* Rhizoma	MOL002157	Wallichilide	42.31	0.71
*Ligusticum striatum* Rhizoma	MOL002140	Perlolyrine	65.95	0.27
*Ligusticum striatum* Rhizoma	MOL000433	FA	68.96	0.71
*Ligusticum striatum* Rhizoma	MOL002151	Senkyunone	47.66	0.24

### 3.2 Compound-target and PPI networks

**Figure 3:**
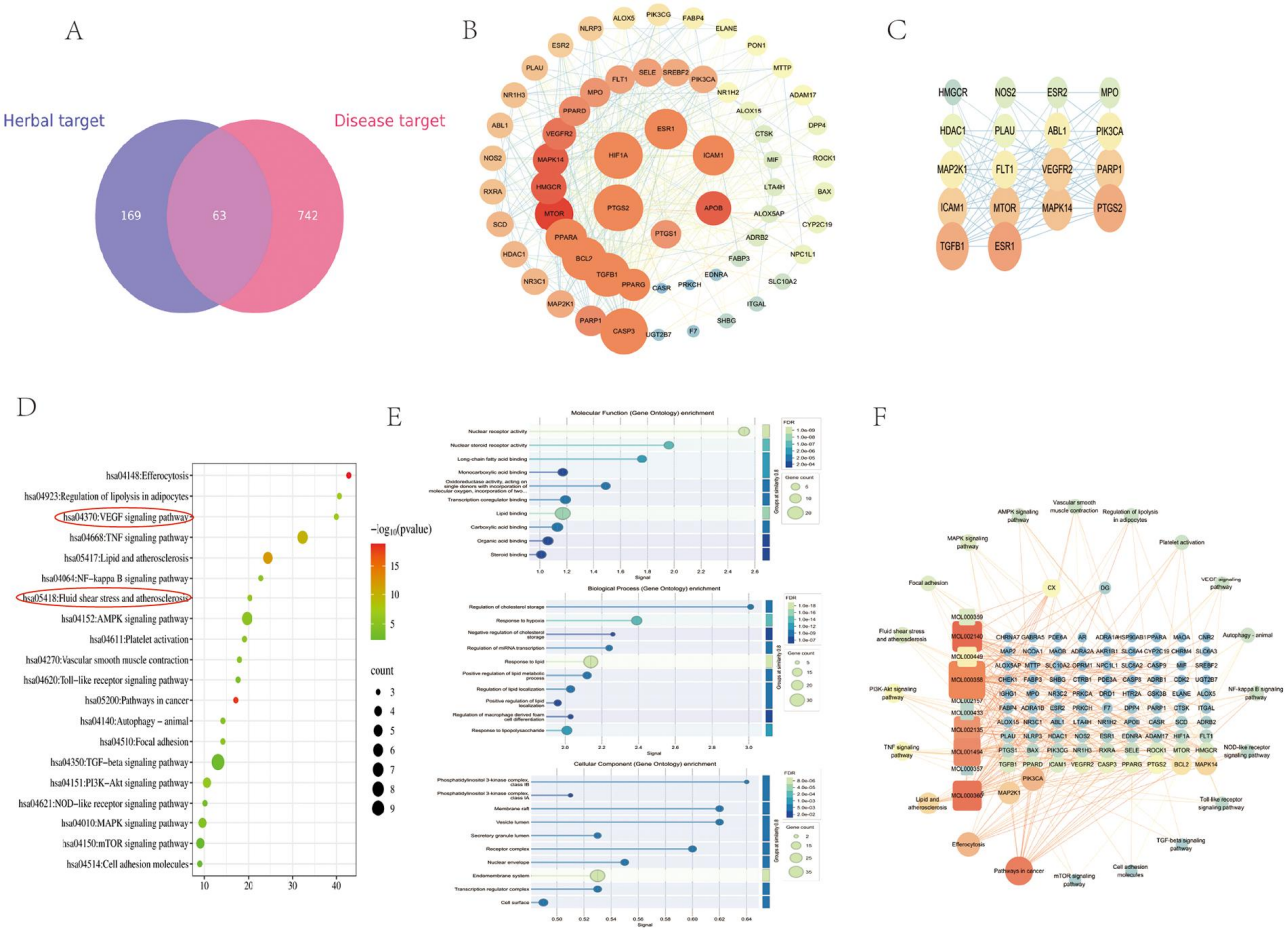
Active-ingredient–target interactions of DG-CX and its correlation with AS-related targets. **(A)** Drug–disease target Venn diagram; **(B)** Protein–protein interaction (PPI) network diagram of Angelica sinensis-Chuanxiong for AS; **(C)** Key submodules of the PPI network; **(D)** KEGG pathway of Angelica sinensis-Chuanxiong for AS; **(E)** GO diagram of Angelica sinensis-Chuanxiong for AS; **(F)** Drug component–pathway–target map. The size and color transparency of nodes represent the degree of value; the larger the area, the darker the color, indicating that the node is more important.

Using the GeneCards, OMIM, DrugBank, and other databases, a total of 805 disease targets of AS were obtained after searching, screening, and de-emphasizing, and 63 common targets were obtained by mapping drug targets to disease targets (Figure 3A). The 63 common targets were subjected to PPI network analysis using the STRING database, and the PPI network was found to contain 63 active nodes and 426 edges, with an average node degree of 13.5. Results from the STRING database were imported into Cytoscape to visualize the PPI network (Figures 3B,C).

### 3.3 GO function and KEGG pathway enrichment analyses

Using the Metascape database, KEGG, and GO analyses were performed on 63 intersecting targets to obtain the relevant pathways and related biological annotations of DG-CX in treating AS. The top 20 entries from KEGG pathway analysis were used for visualization and analysis (Figure 3D), and the top 10 enrichments from GO analysis were used for visualization and analysis (Figure 3E). These targets involved the VEGF signaling pathway, TNF signaling pathway, AMPK signaling pathway, lipids and atherosclerosis, and blood flow shear force and atherosclerosis. Cytoscape was used to construct an herbal medicine–component–target–pathway network diagram (Figure 3F). Findings from network pharmacology revealed that DG-CX could improve AS by modulating vascular endothelial function.

### 3.4 Molecular docking

**Figure 4:**
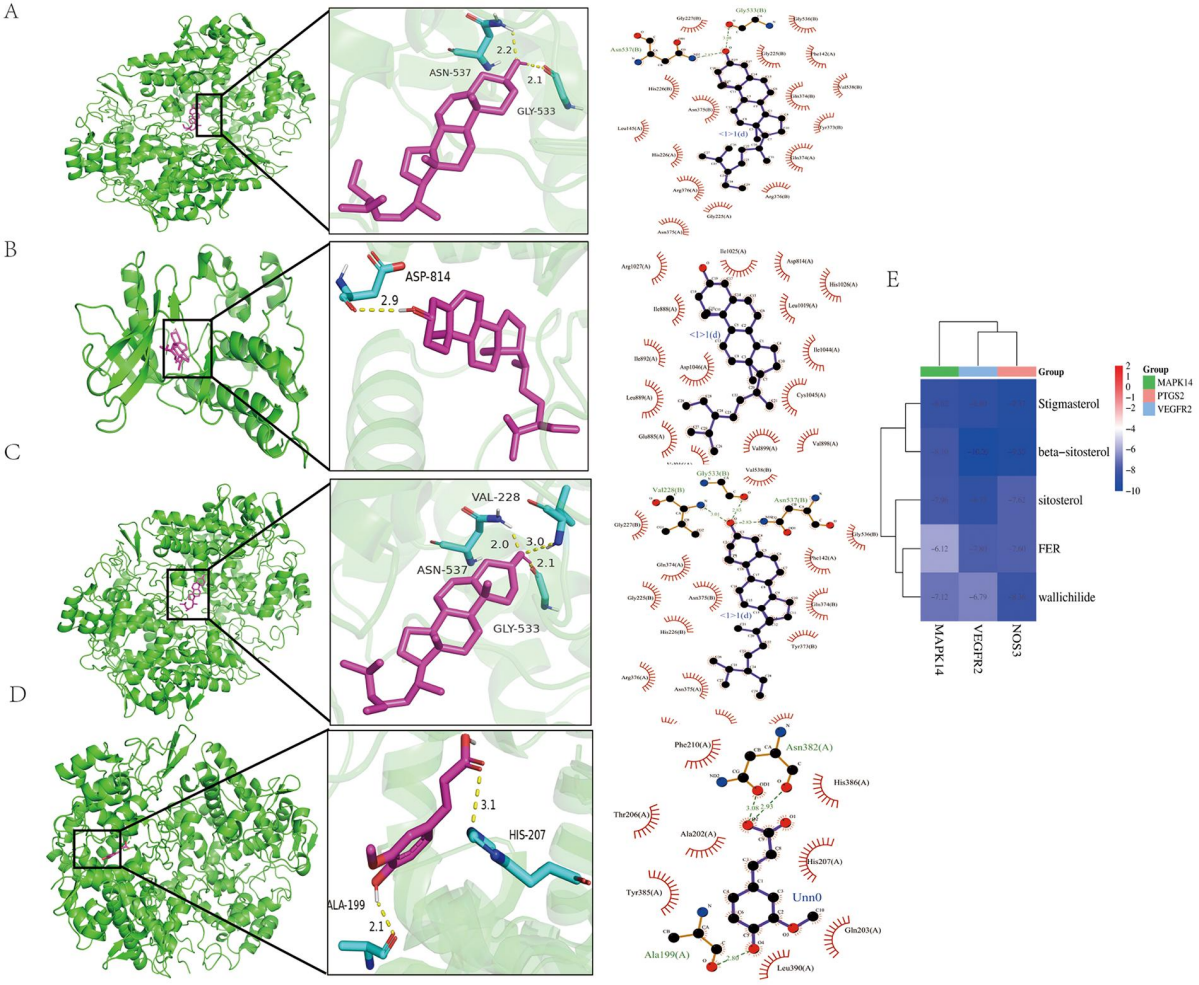
Molecular docking results. **(A)** PTGS2-β-sitosterol; **(B)** VEGFR2-β-sitosterol; **(C)** PTGS2-sitosterol; **(D)** PTGS2-FER; **(E)** Compound-to-target docking results. The larger the absolute value of the negative binding energy of the compound and target binding energy (kcal/mol), the better the docking results of the ligand with the receptor.

Molecular docking enables the further validation of component–target interactions and mechanisms by modeling the affinity ability and binding mode between proteins and ligands (37). The core components of DG-CX were molecularly docked with VEGFR2, MAPK14, and PTGS2, and PyMol was used to visualize the leading results (Figure 4). Molecular docking yielded negative binding free energies for the following complexes: PTGS2–β-sitosterol (–10.82 kcal/mol), VEGFR2–β-sitosterol (–10.05 kcal/mol), PTGS2–sitosterol (–11.04 kcal/mol), and PTGS2–FER (–7.6 kcal/mol), consistent with spontaneous and stable binding. For PTGS2 receptor, β-sitosterol formed hydrogen bonds with ASN537 and GLU533, and hydrophobic interactions with GLY227, HIS226, ASN375, LEU145, ARG376, GLY225, ARG376, GLN374, TYR373, GLN374, VAL538, PHE142, GLY225 and GLY536 (Figure 4A). For VEGFR2 receptor, β-sitosterol formed a hydrogen bond with ASP814 and hydrophobic interactions with ARG1027, ILE888, ILE892, ASP1046, LEU889, GLU885, VAL1916, VAL1898 and CYS1045 (Figure 4B). Sitosterol formed hydrogen bonds (ASN537, GLU533, VAL228) and hydrophobic interactions (GLY227, GLN374, ASN375, GLY225, HIS226, ARG376, LEU145, LEU224, TYR373, PHE142, GLY536) with PTGS2 (Figure 4C). FER formed a hydrogen bond with ALA199 and hydrophobic interactions with PHE210, THR206, ALA202, TYR385, LEU390, GLN203, HIS207, HIS386 on PTGS2 (Figure 4D).

Based on the ranking of the contribution in the PPI, the drug-ingredient–pathway–target map with molecular docking results and the three key genes in the VEGF signaling pathway, namely, VEGFR2, PTGS2, and its downstream factor PGI2, were subjected to *in vivo* experiments for further validation.

### 3.5 Molecular dynamics results

**Figure 5:**
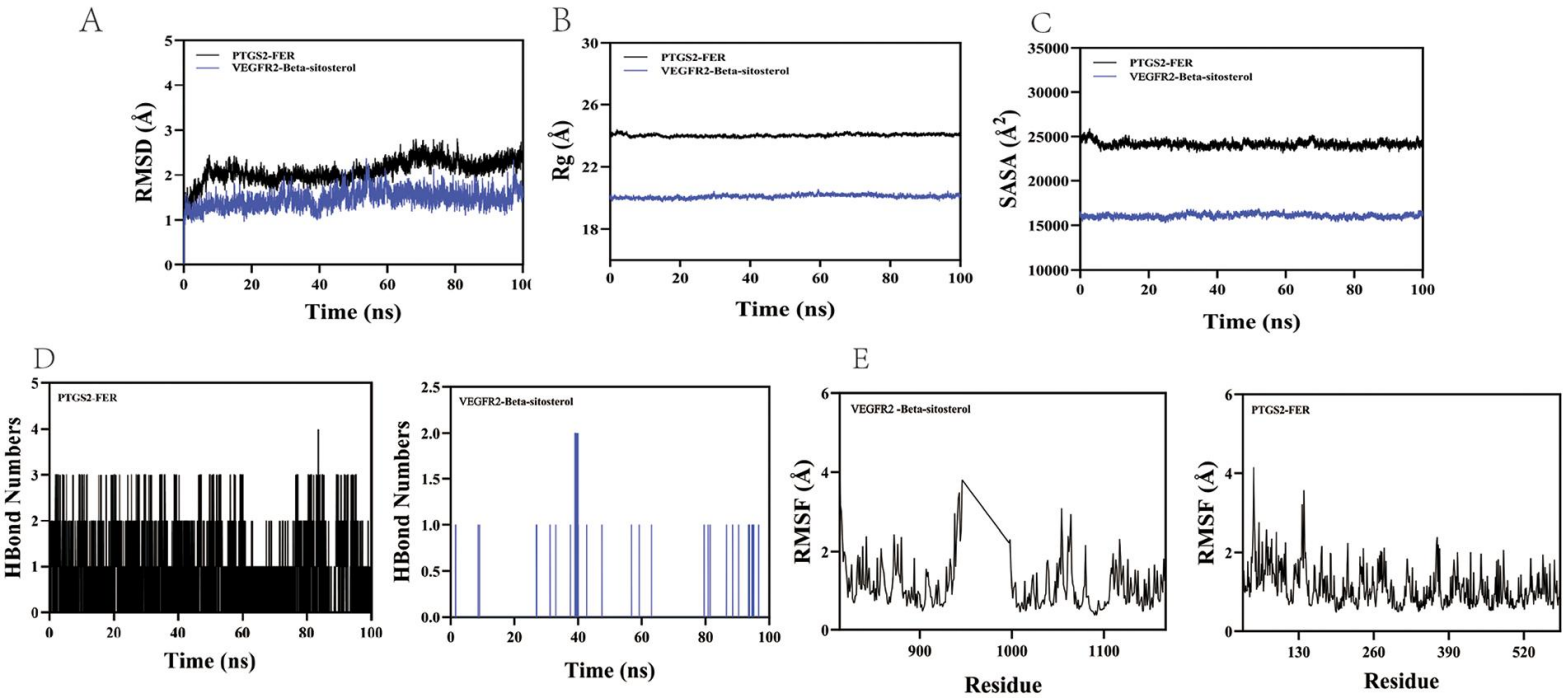
Molecular dynamics simulation results. **(A)** The RMSD values of the protein-ligand complex over time; **(B)** The Rg values of the protein-ligand complex over time; **(C)** The SASA values of the protein-ligand complex over time; **(D)** The HBonds values of the protein-ligand complex over time; **E**. The RMSF values of the protein-ligand complex.

The structural stability and dynamics of the PTGS2-FER and VEGFR2-Beta-sitosterol complexes were assessed through 100-ns MD simulations. The backbone RMSD analysis indicated that both systems equilibrated well: the PTGS2-FER complex stabilized after ∼70 ns with fluctuations around 2.5 Å, while the VEGFR2-Beta-sitosterol complex reached stability earlier (∼40 ns) with a lower deviation of ∼1.7 Å (Figure 5A). The radius of gyration (Rg) remained stable throughout the simulation, suggesting no significant global expansion or compaction in either complex (Figure 5B). Similarly, the solvent accessible surface area (SASA) showed minimal variation upon ligand binding, implying limited perturbation to the protein's overall structure (Figure 5C). Hydrogen-bond interactions were maintained during the simulations. The PTGS2-FER complex formed a median of 2 hydrogen bonds (range 0–4), while VEGFR2-Beta-sitosterol showed a median of 1 bond (range 0–2) (Figure 5D). This consistent H-bond network supports stable ligand binding. Furthermore, residue-wise fluctuations analyzed via RMSF were generally low (<3 Å) for both complexes, reflecting restricted flexibility and high local stability in the binding regions (Figure 5E). In summary, the simulations confirm that both FER and Beta-sitosterol form stable complexes with their respective targets, PTGS2 and VEGFR2, characterized by well-maintained structural compactness, consistent solvent accessibility, and sustained hydrogen-bond interactions.

### 3.6 DG-CX alleviates high-fat diet–induced AS in mice

**Figure 6:**
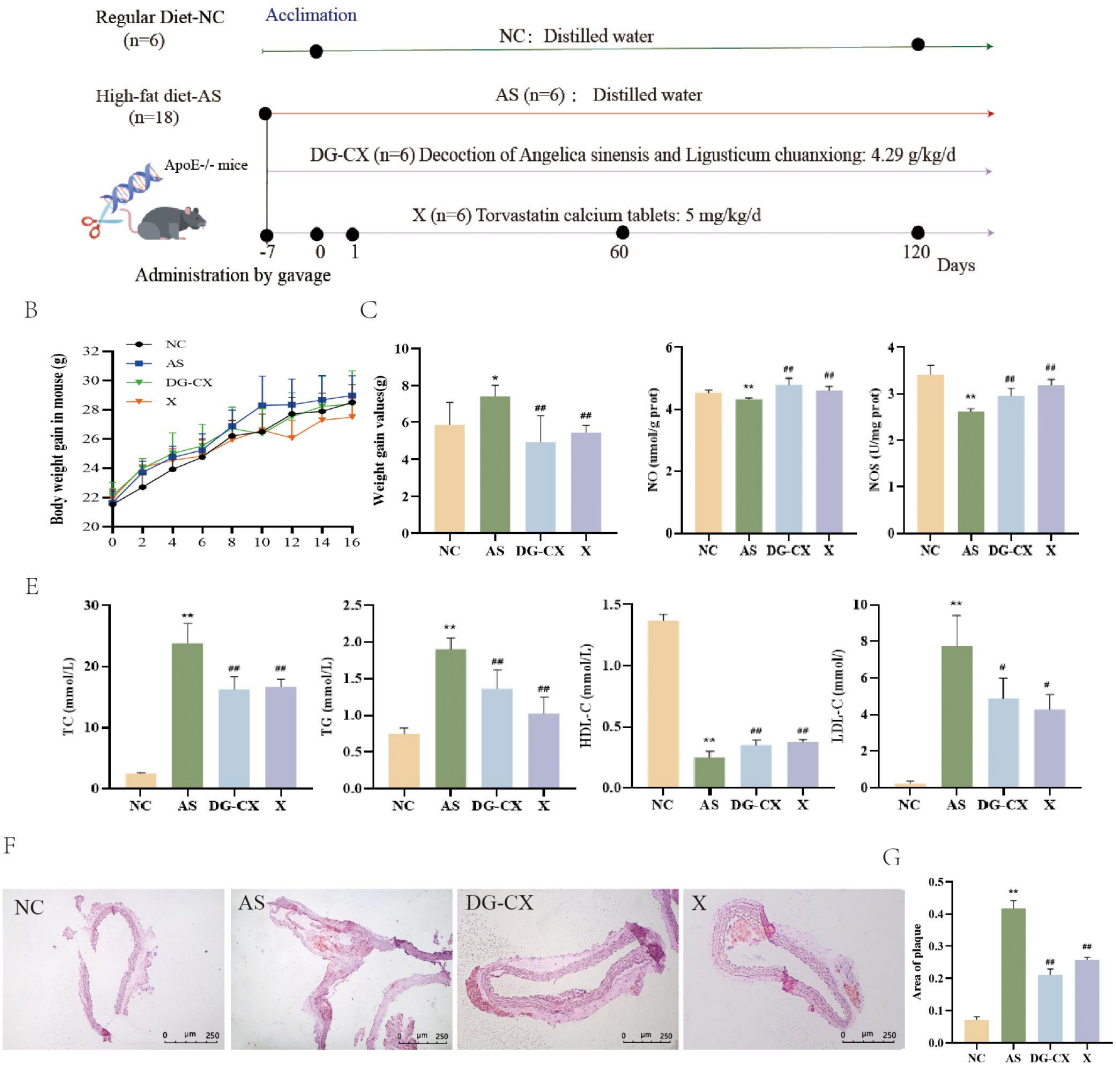
DG-CX alleviates high-fat diet–induced AS in mice (*n* = 6). **(A)** Flow chart of *in vivo* experiments; **(B)** Line graph depicting body weights (mean ± SD); **(C)** Body weight gain; **(D)** Determination of NO and NOS protein levels in aortic vessels; **(E)** Changes in lipid metabolism levels; **(F)** Histopathological findings; **(G)** Vascular plaque area of mice.

Initially, the potential preventive and curative effects of DG-CX were explored using a high-fat diet–induced mouse model of AS. The *in vivo* experimental scheme and study flow is shown in Figure 6A (*n* = 6). Changes in body weight, lipid metabolism, and histopathology of mice were monitored throughout the experiment. The body weights of mice in the AS group increased compared with control mice (*p* < 0.01), and the body weights of mice after DG-CX treatment decreased significantly (*p* < 0.01) compared with those in the AS group (Figures 6B,C, *n* = 6). Disturbed lipid metabolism is one of the pathologic bases for the development of AS. Accordingly, we evaluated the effects of DG-CX on the blood lipid levels of mice. Therefore, we evaluated the effect of DG-CX on the lipid levels of mice. Compared with the AS group, DG-CX and X group could significantly reduce the levels of TC, TG and LDL-C in the serum of mice (*p* < 0.01), while increasing the level of HDL-C (*p* < 0.01), and there was no significant difference between the two *(p* > 0.05) (Figure 6E, *n* = 6).

### 3.7 DG-CX reduces aortic plaque lipid deposition

Histopathological findings did not indicate significant plaque formation in the aortic tissues of mice in the NC group. In contrast, intravascular plaque formation was evident in mice in the AS group, with Oil red O staining confirming prominent lipid accumulation within these plaques. These findings confirm successful AS modeling (*p* < 0.01). Oil red O staining further showed that compared with the AS group, both the DG-CX and X groups could reduce aortic vascular plaques in mice, and the effect of DG-CX was better than that of the X group (*p* < 0.01). This indicates that DG-CX has a significant anti-AS effect (Figures 6F,G, *n* = 6).

### 3.8 Effects of DG-CX on NO and NOS

Endothelial dysfunction is an important reason for the development of AS. The main cause of vascular endothelial dysfunction is decreased NO bioavailability. Therefore, NO and NOS levels in aortic vessels were measured (Figure 6D, *n* = 6). NO and NOS levels in the aortic vessels of mice with AS were significantly reduced compared with those in NC mice (*p* < 0.01 or *p* < 0.05). Compared with the AS group, the levels of NO and NOS in the vascular tissues of the DG-CX and X groups were significantly increased (*p* < 0.01 or *p* < 0.05), but there was no significant difference between them (*p* > 0.05).

### 3.9 Effects of DG-CX on VEGFR2, PTGS2, and PGI2

**Figure 7:**
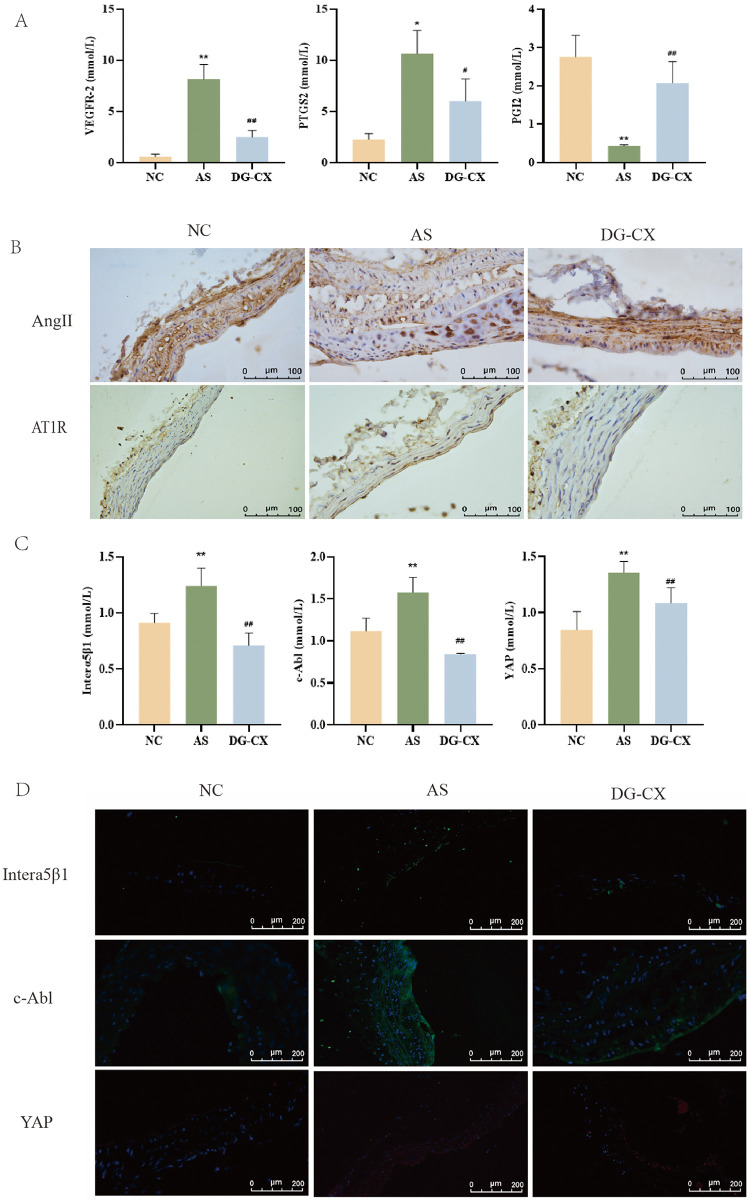
DG-CX protects the vascular endothelium (*n* = 6). **(A)** VEGFR2, PTGS2, and PGI2 mRNA expression in aortic vessels; **(B)** Immunohistochemical staining for AngII and AT1R in aortic vessels; **(C)** interα5β1, c-Abl, and YAP mRNA expression in aortic vessels; **(D)** DG-CX effectively downregulates the c-Abl/YAP signaling pathway to reduce turbulence-associated AS (*n* = 3).

The expression of VEGFR2, PTGS2, and PGI2 was analyzed. The mRNA expression of VEGFR2 and PTGS2 was increased in the AS group mice compared with those in the NC group (*p* < 0.01), whereas the mRNA expression of PGI2 decreased in the AS group mice (*p* < 0.01). In the vascular tissues of the mice aorta, the mRNA expression of VEGFR2 and PTGS2 decreased significantly (*p* < 0.05 or *p* < 0.01), and that of PGI2 increased significantly (*p* < 0.01) after DG-CX intervention (Figure 7A, *n* = 6).

### 3.10 DG-CX protects the vascular endothelium

Furthermore, AngII stimulates the secretion of pro-inflammatory cytokines and enhances vascular smooth muscle cell proliferation and migration, thereby accelerating atherosclerotic plaque formation. AT1R, a key membrane receptor for the action of AngII on vascular walls leading to AS, is widely distributed in various organs. It plays a role in vasoconstriction and promotes endothelial cell proliferation. Therefore, AngII and AT1R levels were analyzed (Figure 7B, *n* = 3). The subendothelial expression of AngII and AT1R increased in mice in the AS group. In contrast, the subendothelial expression of AngII and AT1R was reduced significantly in the aortic vessels of mice after the administration of DG-CX, suggesting its role in improving the function of the endothelium of blood vessels and exerting an anti-AS effect.

### 3.11 DG-CX effectively downregulates the c-Abl/YAP signaling pathway to reduce turbulence-associated AS

Current studies have shown that the c-Abl/YAP turbulence signaling pathway is considered a potential therapeutic target for early AS. Therefore, interα5β1/c-Abl/ YAP was analyzed using PCR and immunofluorescence. The levels of interα5β1, c-Abl, and YAP in the aortic vessels of mice in the AS group were increased compared with those of mice in the NC group (*p* < 0.01); the levels of interα5β1, c-Abl, and YAP mRNA in the aortic vessels of mice reduced significantly after DG-CX intervention (*p* < 0.01 or *p* < 0.05), confirming that the effect of DG-CX in preventing and controlling AS could be achieved by downregulating the c-Abl/YAP turbulence signaling pathway (Figures 7C,D, *n* = 3).

## 4 Discussion

AS is the pathological basis for the development of cardiovascular diseases and the main cause of ischemic heart disease and stroke (38). In recent years, traditional Chinese medicine has shown immense potential for development in treating AS. Traditional Chinese medicines are characterized by their multitarget and multi-action effects as well as high safety and low toxicity (39, 40), all of which can improve lipid metabolism disorders, reduce inflammatory responses, protect the vascular endothelium, and exert anti-AS effects.

Network pharmacology is extensively used to study the complex composition and mechanism of action of Chinese herbs. Network pharmacology analysis of traditional drugs is a common method to study AS and cardiovascular diseases among other disorders (41). DG-CX constitutes a classic pair of Chinese medicine that is widely used to treat hyperlipidemia, coronary heart disease, AS, and other diseases (42, 43). However, only a few studies have conducted molecular docking and *in vivo* experiments to determine the therapeutic effects and mechanisms of DG-CX in AS. Therefore, the combination of network pharmacology and molecular docking, along with *in vivo* experiments, was used in this study to identify the preventive effects and elucidate the pharmacological mechanisms of DG-CX in alleviating AS.

In this study, 10 compounds and their related targets were analyzed using network pharmacology. Blood flow shear force, VEGF signaling pathway, PPAR signaling pathway, and AMPK signaling pathway were found to be likely involved in the mechanism of action of *Angelica sinensis*-Chuanxiong in preventing and controlling AS via KEGG analysis of the 63 targets related to these 10 compounds. Furthermore, the findings from network pharmacology revealed that DG-CX could alleviate AS by improving the vascular endothelium and its related signaling pathways. Its main targets, VEGFR2, PTGS2, and PGI2, may be potential targets in treating AS. Meanwhile, *in vivo* experiments were also performed to verify the results.

VEGFR2 is a major receptor for vascular endothelial cells. It is abundantly expressed on the surface of endothelial cells (44), which are associated with vascular permeability. “Pathological neovascularization” is closely related to the overexpression of VEGFR2, which is phosphorylated, contributing to the proliferation of vascular endothelial cells and activation of endothelial NOS (eNOS), thereby leading to an increase in vascular permeability (45), lipid deposition, and foam cell formation, cumulatively accelerating the development of AS. In this study, DG-CX can down-regulate the expression of VEGFR2, which plays a role in improving vascular permeability and delaying AS.

The PTGS2 gene is also known as cyclooxygenase-2 (COX-2). COX-2 is an inducible pro-inflammatory type that is not expressed in normal arteries (46), and is only seen in atherosclerotic plaques or following specific inflammatory stimuli. Thus, COX-2 is a key factor that is closely related to the development of AS and can be used as a marker to predict the formation and development of AS in a clinical setting (47, 48). Our study shows that DG-CX can down-regulate the expression of COX-2, play a role in inhibiting inflammation and delaying AS. Prostacyclin (PGI2) is a prostaglandin synthesized by endothelial cells. It is mainly produced by vascular endothelial cells and smooth muscle cells under the action of prostacyclin synthetase. PGl2 inhibits leukocyte activation, platelet aggregation, and the proliferation and migration of vascular smooth muscle cells. It also has the effect of dilating blood vessels and can potentially inhibit the progression of AS (49). When the vascular endothelium is damaged, PGI2 levels in the body decrease, and platelet aggregation is aggravated, facilitating the progression of AS and other diseases (50). Our experimental findings confirmed that DG-CX could downregulate the mRNA expression of VEGFR2 and PTGS2, upregulate the mRNA expression of PGI2, and delay the progression of AS.

Recent studies suggest that impaired vascular endothelial function is the initiating link in the development of AS, which is mainly due to decreased NO bioavailability (51). As an endogenous vasodilator, NO can effectively inhibit the expression of inflammatory and adhesion factors, inhibit the migration of smooth muscle cells, and reduce inflammatory infiltration. Furthermore, NO can activate guanylate cyclase, inhibit vascular smooth muscle proliferation, and play a role in vasodilatation (51). eNOS is one of the primary enzymes responsible for NO synthesis. It inhibits lipid peroxidation and the production of reactive oxygen species, preventing the occurrence of AS. Therefore, we determined NO and NOS levels in the vascular tissues of mice. NO and NOS levels decreased in the vascular tissues of mice with AS but increased after the administration of DG-CX, indicating that the drug combination could protect the vascular endothelium and attenuate the process of AS.

AngII is an active component of the renin-angiotensin system. It causes vasoconstriction and promotes an inflammatory response secondary to AS injury (52). AngII participates in the development and progression of AS through inflammatory effects and induces the production of inflammatory factors, including IL-6, CRP, and TNF-α. It promotes the proliferation and migration of smooth muscle cells, leading to the development of AS (53). AT1R is a key membrane receptor for the action of Ang II on vascular walls, which leads to AS after activation of the renin-angiotensin-aldosterone system. This membrane receptor is widely distributed in various organs, acting as a vasoconstrictor and promoting endothelial cell proliferation. AT1R induces the protein expression of monocyte chemoattractant protein-1 by promoting monocyte and macrophage aggregation and smooth muscle cell migration and proliferation, and also participates in the development of AS (54). Therefore, AngII and ATIR in the vascular tissues of mice were analyzed. DG-CX intervention decreased AngII and ATIR levels in vascular tissues, suggesting its efficacy in inhibiting vasoconstriction and exerting anti-AS effects.

Furthermore, studies have found that abnormal blood flow shear stress can promote inflammatory responses, activate related inflammatory signaling pathways, upregulate the expression of factors such AS adhesion factors, promote the activation of YAP in the nucleus, and promote the development of AS (55). In our previous study, we also found that Danggui Shaoyao Powder can regulate the c-Abl/YAP turbulent signaling pathway and exert an anti-as effect. Therefore, we consider c-Abl/YAP to be a potential therapeutic target for the prevention and treatment of early AS. In this study, we found that DG-CX can indeed down-regulate the expression of Interα5β1, c-Abl and YAP mRNA, which is consistent with the immunofluorescence results, indicating that DG-CX may be a potential effective drug for the prevention and treatment of AS. However, This study utilized network pharmacology to predict potential compounds for DG-CX in treating AS, conducted preliminary screening and explored the underlying molecular mechanisms. Further extensive experiments are required to provide empirical evidence. Our research is limited to animal experiments. The animal models are relatively simplistic and may have certain differences from human atherosclerosis. Moreover, the dosage administered is uniform. These factors increase the difficulty in promoting the application of DG-CX in clinical trials. Therefore, future research will focus on verifying its effectiveness in higher-level preclinical models and systematically evaluating its pharmacokinetic characteristics and long-term safety, thereby facilitating the transition of DG-CX to clinical application.

## 5 Conclusions

The complex pathogenesis of atherosclerosis involves the intricate interaction among inflammatory responses, dyslipidemia, and vascular endothelial injury. Treatments targeting a single pathological mechanism are often insufficient to reverse the progression of AS. In contrast, DG-CX may exert multi-target therapeutic effects by simultaneously regulating inflammatory responses, vascular endothelial function, and the C-Abl turbulent signaling pathway. However, the specific correlation between them still awaits further experimental verification.

## Data Availability

The original contributions presented in the study are included in the article/Supplementary Material, further inquiries can be directed to the corresponding authors.
